# Implementation of artificial intelligence and non-contact infrared thermography for prediction and personalized automatic identification of different stages of cellulite

**DOI:** 10.1007/s13167-020-00199-x

**Published:** 2020-02-07

**Authors:** Joanna Bauer, Md Nazmul Hoq, John Mulcahy, Syed A. M. Tofail, Fahmida Gulshan, Christophe Silien, Halina Podbielska, Md. Mostofa Akbar

**Affiliations:** 1 Department of Biomedical Engineering, Faculty of Fundamental Problems of Technology, Wrocław University of Science and Technology, Wybrzeze Wyspiańskiego 27, 50-370 Wrocław, Poland; 2 Department of Physics and Bernal Institute, University of Limerick, Limerick, Ireland; 3 Department of Computer Science and Engineering, Bangladesh University of Engineering and Technology, Dhaka, Bangladesh; 4 Department of Materials and Metallurgical Engineering, Bangladesh University of Engineering and Technology, Dhaka, Bangladesh

**Keywords:** Cellulite, Artificial intelligence, Infrared thermography, Predictive preventive personalized medicine, Prediction and health monitoring

## Abstract

**Background:**

Cellulite is a common physiological condition of dermis, epidermis, and subcutaneous tissues experienced by 85 to 98% of the post-pubertal females in developed countries. Infrared (IR) thermography combined with artificial intelligence (AI)-based automated image processing can detect both early and advanced cellulite stages and open up the possibility of reliable diagnosis. Although the cellulite lesions may have various levels of severity, the quality of life of every woman, both in the physical and emotional sphere, is always an individual concern and therefore requires patient-oriented approach.

**Objectives:**

The purpose of this work was to elaborate an objective, fast, and cost-effective method for automatic identification of different stages of cellulite based on IR imaging that may be used for prescreening and personalization of the therapy.

**Materials and methods:**

In this study, we use custom-developed image preprocessing algorithms to automatically select cellulite regions and combine a total of 9 feature extraction methods with 9 different classification algorithms to determine the efficacy of cellulite stage recognition based on thermographic images taken from 212 female volunteers aged between 19 and 22.

**Results:**

A combination of histogram of oriented gradients (HOG) and artificial neural network (ANN) enables determination of all stages of cellulite with an average accuracy higher than 80%. For primary stages of cellulite, the average accuracy achieved was more than 90%.

**Conclusions:**

The implementation of computer-aided, automatic identification of cellulite severity using infrared imaging is feasible for reliable diagnosis. Such a combination can be used for early diagnosis, as well as monitoring of cellulite progress or therapeutic outcomes in an objective way. IR thermography coupled to AI sets the vision towards their use as an effective tool for complex assessment of cellulite pathogenesis and stratification, which are critical in the implementation of IR thermographic imaging in predictive, preventive, and personalized medicine (PPPM).

**Electronic supplementary material:**

The online version of this article (10.1007/s13167-020-00199-x) contains supplementary material, which is available to authorized users.

## Introduction

Both artificial intelligence (AI) [1] and infrared (IR) thermography [2] are promising tools to be used in predictive, preventive, and personalized medicine (PPPM) in terms of fast and better diagnosis of diseases and more objective understanding of disease progress [3]. The implementation of personalized medicine based on AI algorithms is still in its early stages. The advancement of IR imaging, especially the availability of IR camera in mobile phones, can stimulate development and application of smart phone “app”-based personalized diagnoses especially for prescreening. The success of implementation of AI in IR thermography-based diagnosis will need clinical validation for accuracy and predictability, which is an essential feature for PPPM. This has prompted us to implement AI in IR thermography-based recognition in properly controlled clinical setting in the case of cellulite identification.

### Cellulite and its staging

Cellulite, also known as gynoid lipodystrophy, edematous fibrosclerotic panniculopathy, adiposis edematosa, dermopanniculosis deformans, status protrusus cutis, or orange peel syndrome, is one of the most common, aesthetic dermis, epidermis, and subcutaneous tissue conditions, which appears as dimpled skin [4]. Cellulite is considered in the literature as a cosmetic defect, not as disease or disorder. It should not be confused with cellulitis, which is an infectious disease caused by bacteria and involves the inner layers of the skin [5].

Cellulite manifests as the herniation of subcutaneous fat within fibrous connective tissue, which can be often observed for example in the backsides of the pelvic region, lower limbs, and abdomen [6]. It sometimes occurs in the breast region, lower part of the abdomen, upper arms, the nape region of neck and areas, in which the female pattern of adipose deposition can potentially take place. Cellulite occurs in 85 to 98% of post-pubertal females [7, 8]. First symptoms of cellulite may appear as early as during adolescence. It can affect about 12% of adolescent girls. Appearance of cellulite increases significantly (by about 20%) during pregnancy due to the higher supply of female sex hormones. Cellulite is also prevalent in high proportion (nearly 25%) in menopausal or perimenopausal women due to a decline in steroid concentrations and water management disorders [9]. Women of all ethnicities can have cellulite [10] but the prevalence is more common in Caucasian females than in Asian females [11]. It is rarely seen in males [12] except for those with androgen-deficient states such as Klinefelter’s syndrome, hypogonadism, post-castration states, and patients receiving estrogen therapy for prostate cancer.

Cellulite can affect physical beauty of women, negatively impact women’s self-esteem, and derogate their quality of life [13]. It is important to devise tools and protocols for early prediction and/or diagnosis of different stages of cellulite that can aide personalized medicine to treat cellulites. Such predictions and diagnoses need to be objective, quantitative, and automatic. It will then become easy for individuals to test their stages of cellulite repeatedly and reliably on their own and/or with the help of a professional cosmetologist or dermatologists.

**Fig. 1:**
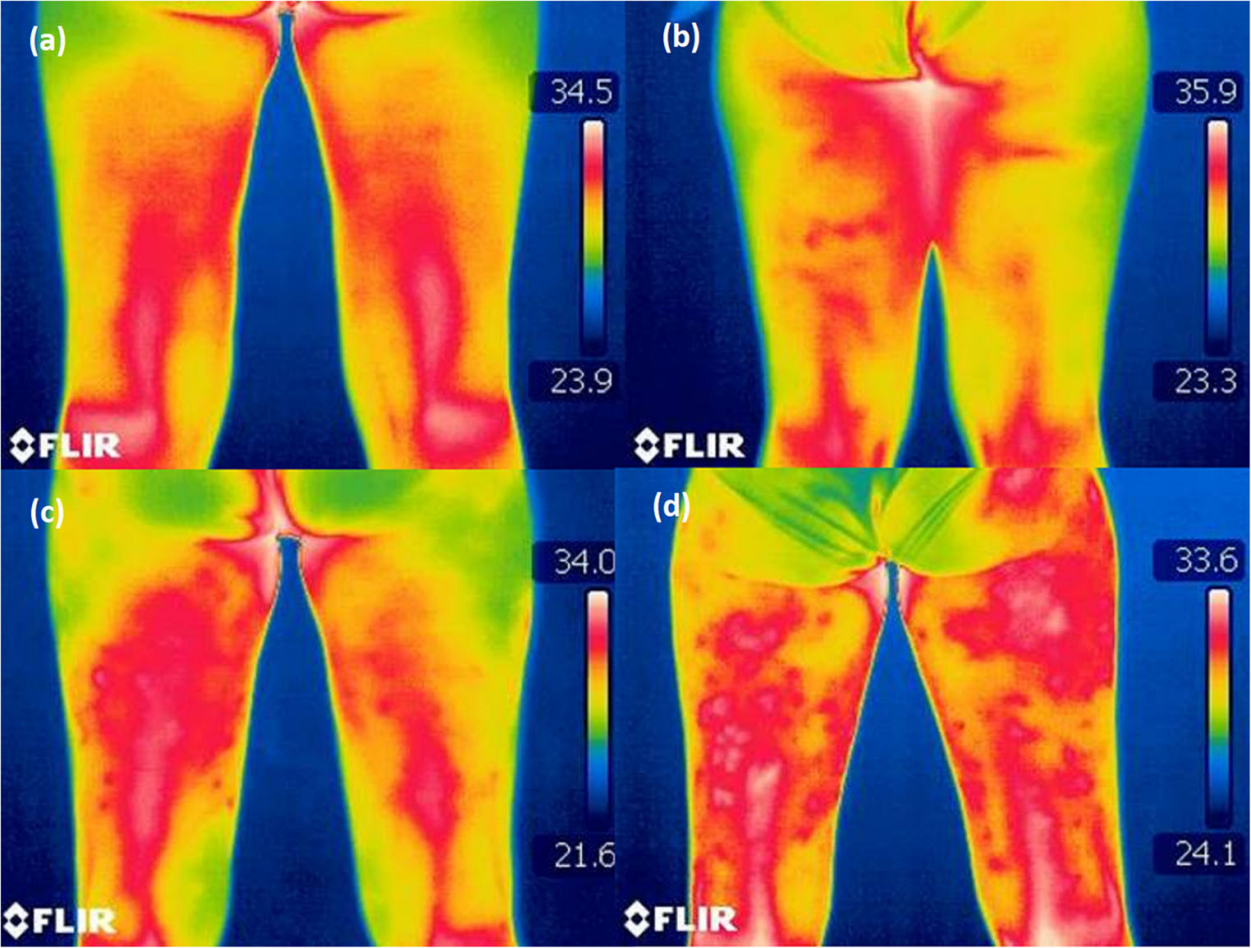
Thermographic images of healthy skin and subsequent stages of cellulite diagnosed by means of the Nürnberger-Müller scale. (**a**) Stage 0—cellulite-free skin. (**b**) Stage 1—mild cellulite. (**c**) Stage 2—moderate cellulite. (**d**) Stage 3—severe cellulite

Cellulite develops in a few overlapping stages, which, to fully manifest at a clinically diagnosable level, can take months and sometimes even years (Fig. 1). Depending on the consistency of the skin, cellulite can be classified into four general types, namely hard, soft, edematous, and mixed (overlapping). Hard cellulite occurs in young women. Physically slim women with relatively tight and firm skin are more prone to hard cellulite, where dimples appear only during a change in the body position or when the skin is pinched. Hard cellulite can transform into the so-called soft cellulite over time. Soft cellulite is usually found in mature women with low physical activity. Loss of muscle mass, strength, and tone (hypotonia) and an increase in the fat volume lead to soft cellulite, which is characterized by a progressive loss of elasticity, pliability, and flaccidity of skin [14, 15]. In this stage, irregular beads and nodules can appear. They may also cause pain. Edematous cellulite stage is relatively rare and manifests itself with a significant increase in the volume of the lower limb tissues. In this stage, the patient experiences a severe feeling of heavy and sore legs. A positive result of Godet’s test, which accompanies edema and manifests as dimple in the skin when pinched, generally characterizes this stage of cellulite [6]. Mixed cellulite is more common in women who already have different types and stages of cellulite at different locations of their body.

### Non-contact infrared thermography for cellulite diagnosis

Studies of the physiology and anatomy of adipose tissue including cellulite disorder usually follow two approaches, firstly in vitro studies of isolated adipocyte and secondly in vivo studies with noninvasive or minimally invasive functional methods [16, 17]. In vitro studies need an expert operator and direct contact of subjects to diagnose the stage of cellulite. For example, contact thermography has been used for cellulite diagnosis, where specialized liquid crystal mats are required to be applied to the suspect site of the body [18]. IR thermography, on the other hand, does not require any direct contact with the patient and allows remote and noninvasive assessment of cellulite based on the surface temperature distribution [19, 20]. It is also safe, painless, and relatively inexpensive. Infrared thermography is commonly used in vivo approach applied for detection of different diseases like tumors [21–24]. Thermal temperature mapping has also been used in skin lesion differentiation [25] and rosacea pattern detection [26]. It is also used for the measurement of distribution of superficial temperature for inter alia screening tests [27], diagnosis [28], treatment or performance assessment [29–31], physiotherapeutic procedure evaluation [32–34], personalized medicine [2], and prevention [35, 36].

### Computer-aided methods and artificial intelligence in the classification of cellulite stages

Several groups have put forward computer-based classification systems for diagnosis of cellulite from IR thermography [2, 9, 37, 38]. Most of these studies involved manual image preprocessing and feature extraction methodologies. Advanced machine learning algorithms, such as artificial neural network (ANN), have been also used to classify cellulite stages; however, the achieved accuracy was rather unsatisfactory ~ 70% [37]. Recently, the application of artificial intelligence was reported in case of detection and prevention of other dermatological diseases [39–42]. The selection of appropriate features and classification algorithm was considered a critical determinant of a classification system as it defines the accuracy of the final diagnosis through classification accuracy, algorithm performance, availability of data, and resource constraints [43, 44].

### The role of information technologies and artificial intelligence for development of predictive, preventive, and personalized medicine

In this article, we describe the application of advanced information technologies, e.g., artificial intelligence, to aid in the diagnosis involving IR imaging. This approach may become hugely important in near future in contemporary medicine, especially in promoting the cutting-edge patient-oriented, so-called P3, paradigm that seeks to make medicine more predictive, preventive, and personalized (PPPM) [3, 45]. The term “personalized medicine” refers to the patient-tailored therapy that maximizes efficiency of the treatment based on the individual profile of a person. It can also refer to a stratified medicine approach, which lies somewhat in between the standardized medical procedures and fully individualized approach. The stratification of large patient groups into subpopulations according to some characteristic features is indeed the first step towards person-centered medicine. Such an approach makes it possible to introduce individualized treatment for patients falling within a given stratified group. The treatment can be adapted to the entire stratified group and may lead towards a much better medication efficacy [46, 47].

Information and communication technologies (ICT) can become not only a very important means but also a prerequisite for successful implementation of PPPM. ICT, including technology proposed by our approach, may facilitate implementing the PPPM by providing necessary tools, for example, for disease modeling (e.g., prediction of a disease development, disease relational models, process models) [48], patient stratification among big populations [49], planning optimized, less invasive diagnostic approaches [50], remote patient health state and therapy outcomes monitoring [51], development of the patient-oriented therapy algorithms [52], or even creation of an individual patient profile which leads to individually tailored therapy [53]. We observe from this literature and our own assessment of both current and emerging medical markets that the importance of ICT will soon become greater and may even radically impact the healthcare sector. This is due not only to the very rapid development of new technologies that can be translated to medicine but also due to the changes in the perceived role of ICT in medicine. In particular, it can be expected that computer savvy young and middle age people will be much more open to, for example, ICT-based diagnosis tools because of their adoption of modern technologies, which they see as an indispensable part of the life.

The importance of ICT for the further development of PPPM is emphasized not only by the growing number of publications in recent years, but also by completely new applications. Lemke et al. [54] recently underlined the role of information technology for collection, organization, and utilization of medical information. Such medical information may include genomics, proteomics data, and patients’ imaging records along with laboratory tests’ results and doctors’ assessment. This requires the analysis of vast amounts of personal health data. Also Schork [55] has recently highlighted the role of AI in storing, aggregating, accessing, and integrating the medical data. They point out unique advantages of AI for design and implementation of appropriate intervention targets and strategies for patient-oriented individualized treatment. We feel that progress in PPPM would be highly dependent on the development of various ICT tools used in prediction, diagnosis, and personalized treatment. Information technology may allow tailoring and customizing treatment of multiple diseases, depending on a patient’s physiological profile and lifestyle. This requires the analysis of numerous biomarkers and data recorded on microscopic and macroscopic levels. Our current study should be considered as a first step in that direction.

## Materials and methods

### Materials

**Fig. 2:**
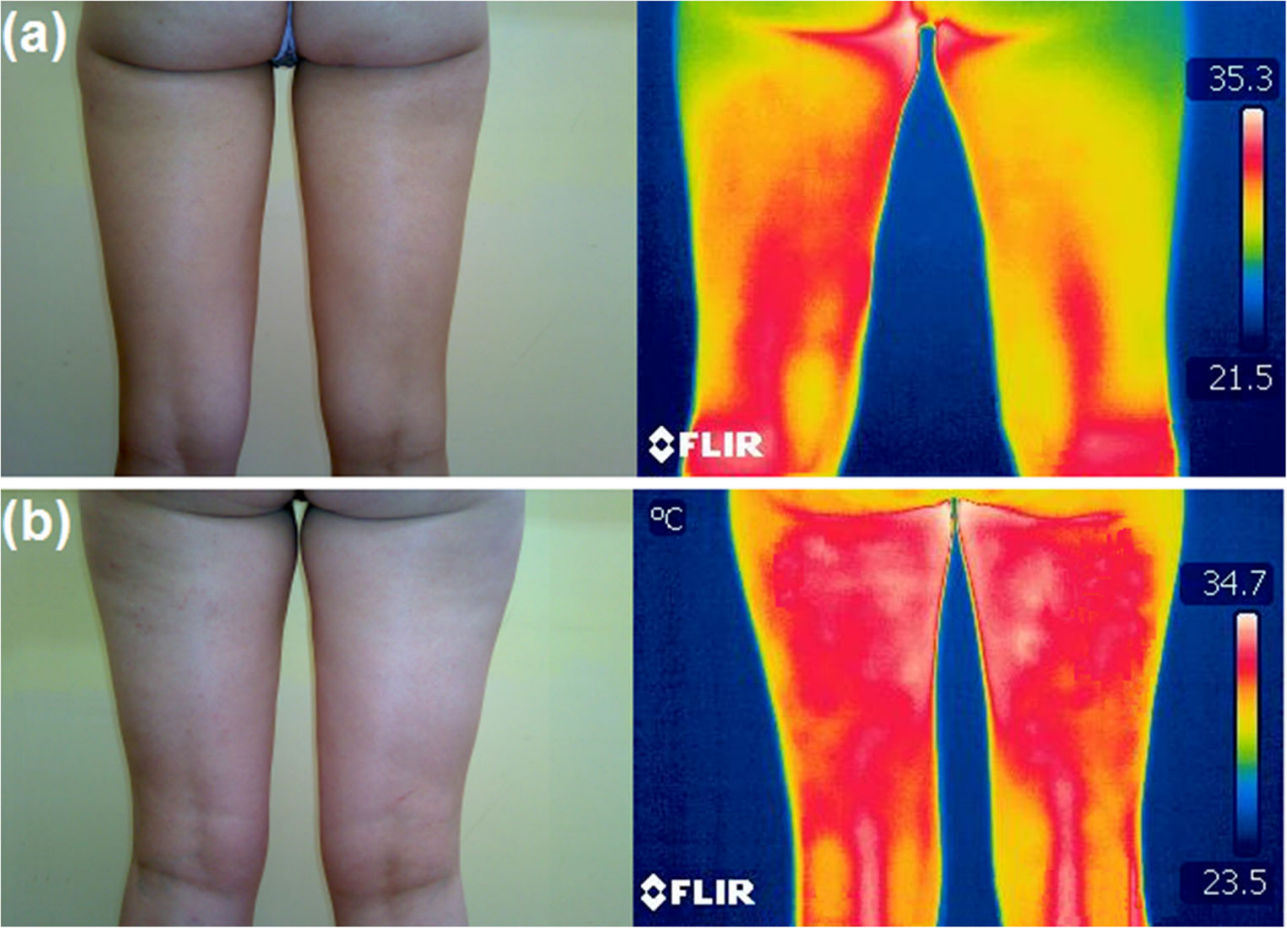
The visual and thermographic images of healthy volunteer (**a**) and volunteer with severe cellulite (**b**)

The experiment was conducted on total 212 female volunteers maintaining the ethical guidelines of the Declaration of Helsinki and following the approval of the Senate Ethics Committee for Scientific Research at the University School of Physical Education in Wrocław. Collected thermal image resolution was 320 × 240 pixels. These images were taken under informed written consent from the patients with different stages of cellulite. All recommendations for thermographic measurements in medical applications, including the equipment requirements, as well as an experiment preparation and its carrying out were rigorously followed [56]. To minimize any external environmental effects, the measurements were carried out in the dedicated experimental room with constant ambient conditions. Both the room temperature and humidity were monitored (respectively 22–24 °C and 35–40%). The thermographic camera FLIR T335 operating at a spectral range of 7.5–13 μm with a temperature sensitivity of 50 mK at 30 °C was applied for all measurements. The thermographic images of posterior part of the volunteers’ thighs were recorded in standing positions after 20 min of the preliminary adaption. Images were taken at a fixed distance of 1.2 m. Before thermogram recording, a licensed cosmetologist has evaluated the severity stage of the cellulite for all volunteers in accordance with the Nürnberger-Müller scale (Table 1). Information about skin condition was coded in the file name. The distributions of training and testing datasets from thermographic images throughout different stages of cellulite are shown in Table 2. Figure 2 compares typical, visible light, and thermographic images of a volunteer with healthy skin and cellulite.

**Table 1:** Nürnberger and Müller scale for classification of cellulite severity

Cellulite stage	Clinical characteristics
0	Healthy skin, no skin surface alterations
1	Mild cellulite, the skin surface is smooth while the person is standing or lying. The alterations appear after pinching
2	Moderate cellulite, the so-called orange peel or mattress appearance is evident without pinching when standing
3	Severe cellulite, skin alterations are evident in both, standing and lying position

**Table 2:** Distribution of training and testing dataset from thermographic images among different stages of cellulite

Stage	Number of images in the database	Number of training data	Number of test data
Stage 0 (healthy)	27	19	8
Stage 1 (mild)	93	65	28
Stage 2 (moderate)	61	43	18
Stage 3 (severe)	31	22	9
Total	212	149	63

### Thermographic images preprocessing

After the acquisition, all images went through a preprocessing step. For morphological analysis, we want to explore only the area that is responsible for cellulite. So we remove the temperature bar and the text from the image. We built an algorithm (more detailed description in supplemental materials: Appendix 1) to automatically crop the image and select only the region of interest (ROI), which is the lower thigh region. We first increase the contrast of the image for easy detection of the cellulite area by adjusting image histogram. Then using Sobel’s transformation, we detect the edges of the images and the thighs. Next, we remove noise and finally crop the image. All images are resized to 210 × 240 pixels from 320 × 240. Finally, we convert the RGB colored images to grayscale images for feature extraction.

### Classification of cellulite severity stage

All experiments carried out for the purpose of this study were performed with use of Matlab 17 (MathWorks Inc). We applied pattern recognition (PR) to enable personalized, fast, accurate, and early diagnosis of cellulite and objective evaluation of its severity stages. PR is an engineering application of machine learning which is a branch of artificial intelligence (AI). Machine learning deals with the design and building of systems that can learn from data, whereas PR enables automatic classification of data based on previously gained knowledge. The PR approaches used here are able to automatically learn and then recognize unknown images. The learning process was provided based on a learning/training database and then validation of the systems accuracy was done with use of independent test database.

**Fig. 3:**
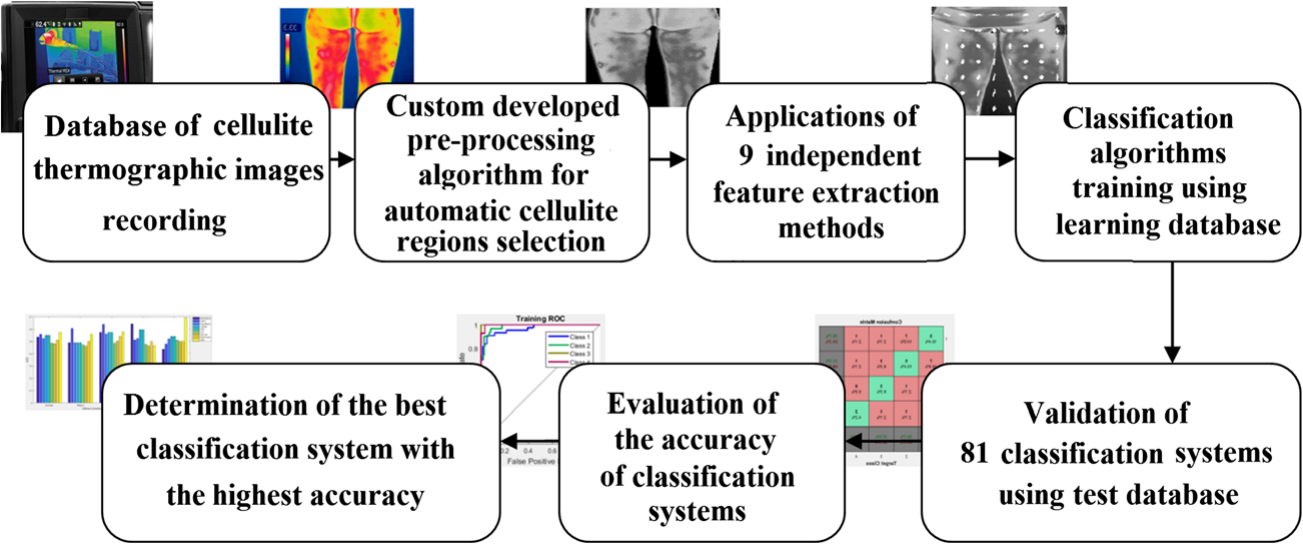
Automatic cellulite classification protocol for IR thermographic images of cellulite

For recognition of different stages of cellulite, we developed a multiclass classifier. During the systems’ learning, the training dataset as an input for the classification algorithms was used. Classification algorithms use the training data, tune the parameter when needed, and build a classification model. In the training step, a positive (1) or negative (0) result is assigned to each dataset which indicate whether the dataset belongs to a particular stage. As our datasets are not balanced over different stages (please see Table 2), we use binary classification for each stage and then take the best probability score as the output stage. In testing step, we used the test dataset to check the correctness (performance) of the classification model. The full process of cellulite image recognition is shown in Fig. 3.

**Fig. 4:**
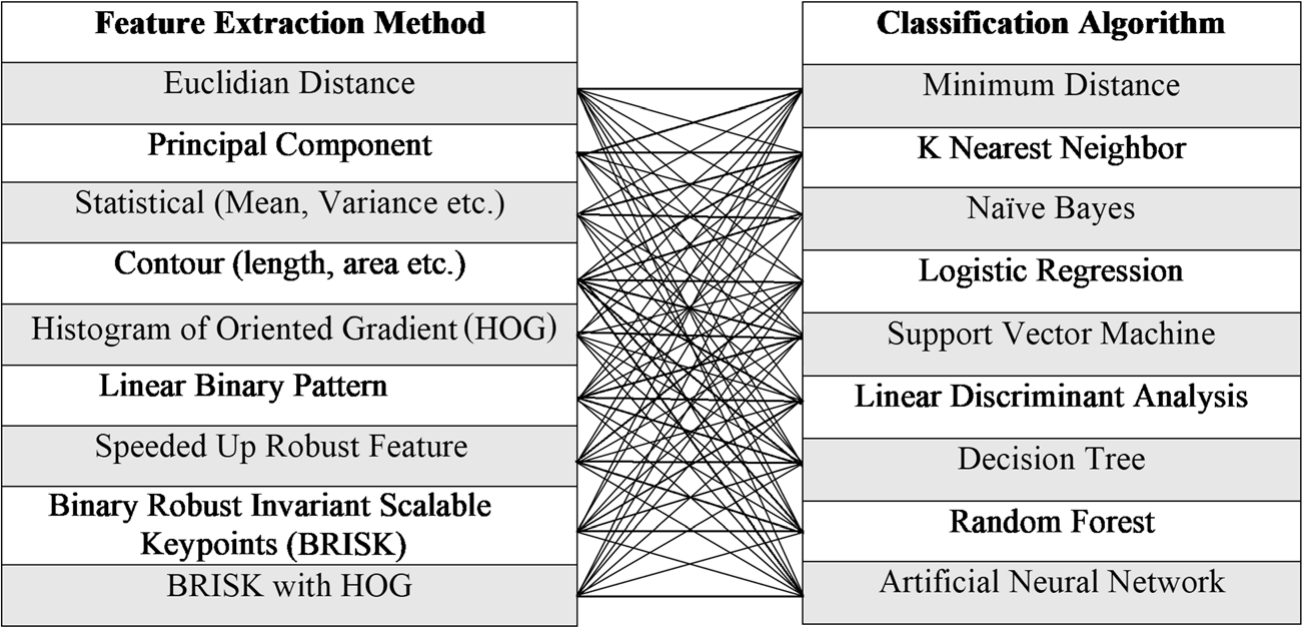
Combination of feature extraction methods and classification algorithms applied. For each of 9 applied feature extraction methods, all the classification algorithms were used to observe the differences in the performance. Similarly, for one classification algorithm, all the 9 feature extraction methods were tested to observe the differences in performance of feature extraction methods

Altogether, the 81 different recognition systems were built and tested for the purpose of this study. We have used 9 feature extraction methods to learn our systems and then combined each of them with 9 different classification algorithms in order to choose the best combination. The methods used and their combinations are specified in Fig. 4.

After the analysis of performance results of 81 models, we chose the histogram of oriented gradient (HOG) for extracting the features from images. HOG uses the distribution (histograms) and directions of oriented gradients as the feature and encodes local shape information from regions within an image. We experimented with different HOG cell sizes and found that 48 × 48 cell size provides the best results. In total, we got 432 features from HOG for each image, whereas the original preprocessed image has dimension of 210 × 240, which gives a total of 50,400 features.

**Fig. 5:**
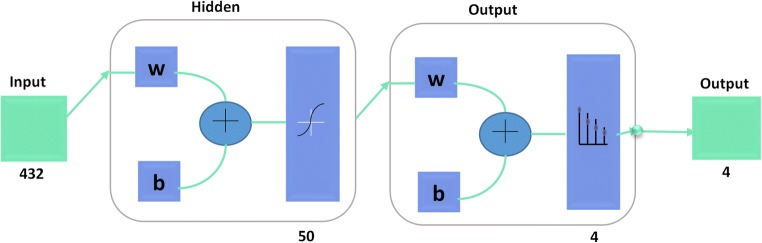
Architecture of ANN used for cellulite stage classification. The ANN is a three-layer perceptron. The information is sent in one direction, from the input nodes, through the hidden nodes, to the output nodes. Gradient descent algorithm is used for back propagation

Finally, we chose multilayer feed forward artificial neural network (ANN) for classification purpose as the HOG and ANN combination gives the best results among of all 81 tested models. The architecture of the used ANN is shown in Fig. 5.

### Accuracy measures of tested classification models

For each tested classification model, efficacy measures such as sensitivity (true positive rate (TPR)), specificity (true negative rate (TNR)), precision (positive predictive value (PPV)), and *F*-score value were calculated. Furthermore, the fall out (false positive rate (FPR)) and miss rate (false negative rate (FNR)) errors were measured. To check the quality of classifiers, we have also plot the receiver operating characteristics (ROC) and calculated the area under the curve (AUC). The AUC can range from 0 to 1. The more convex ROC and higher AUC value, the better accuracy of the proposed model. ROC and AUC help to compare the performance of applied classification models for different cellulite stages, as well as show the discrepancy between the performance due to the quality and size of the available data.

## Results

### Automatic extraction of region of interest areas

**Fig. 6:**
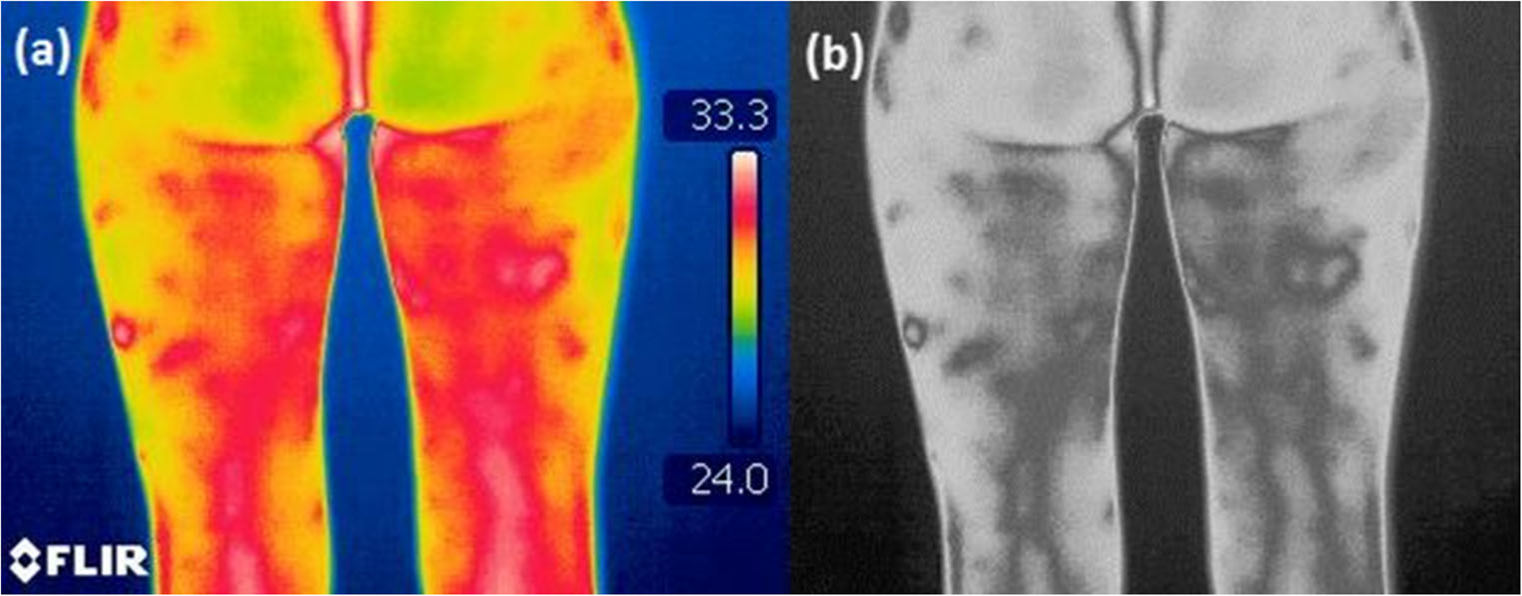
Automatic extraction of region of interest (ROI) area during image preprocessing. (**a**) Original thermographic image. (**b**) Preprocessed image of posterior site of thigh region

We have developed an algorithm to automatically detect and extract the ROI, namely the posterior site of thighs (for more details please see the supplemental materials: Appendix 1). With noise removal and fine tuning of our algorithm, we successfully extracted the posterior thigh region from all 212 images. An example of automatic extraction of ROI is shown in Fig. 6. This preprocessing converts an original 320 × 240-pixel color thermographic image to a 210 × 240-pixel grayscale image.

### Validation of the classification algorithms

For validating the classification algorithms and evaluating their performance, we have divided our clinical thermographic data into two independent and separable sets: training and test dataset. We avoided any random selection of training dataset as such approach may lead towards weak representation of a given stage of cellulite if a small number of datasets are selected for that stage. We decided to use 70% of the image data of each class for training and the remaining 30% of the data for testing the performance of the classification algorithm, what ensures the presence of each dataset in the training set. The distribution of training and test datasets are shown in Table 2. We use multiclass approach to classify different stages of cellulite. Binary classification was performed for each of the stages and the results providing maximum probability score were selected.

We have used ANN with 9 feature extraction methods such as the following: principal component analysis (PCA), Euclidean distance (EucDist), statistical feature (Stat) of intensity value of the image (mean, variance, skewness, etc.), contour-based feature (Cont), i.e., length of contour, area of contour, HOG, linear binary patterns (LBP), speeded up robust feature (SURF), binary robust invariant scalable keypoints (BRISK), and combination of BRISK with HOG (HOG_BRSIK). The details of applied features’ extracted methods and associated results are described in supplemental materials (see Appendix 2 and Appendix 3: Tables S1-S4). Among the feature extraction methods, the HOG performed the best with an average AUC of 0.77 and accuracy 80.9%.

In the next step, HOG was combined with 9 different classification algorithms to find an identification system providing the higher performance. Such classifiers as minimum distance (MinDist), *K* nearest neighbor (KNN), naive Bayes (NB), logistic regression (LogReg), support vector machine (SVM), linear discriminant analysis (LDA), decision tree (DT), random forest (RF), and ANN were tested. In each case, the classifying systems were trained with use of the training datasets described in Table 2 and then validated with the independent test datasets. The accuracy results for combination of HOG and different classification algorithms are shown in supplemental materials (see Appendix 4: Tables S5-S8).

**Fig. 7:**
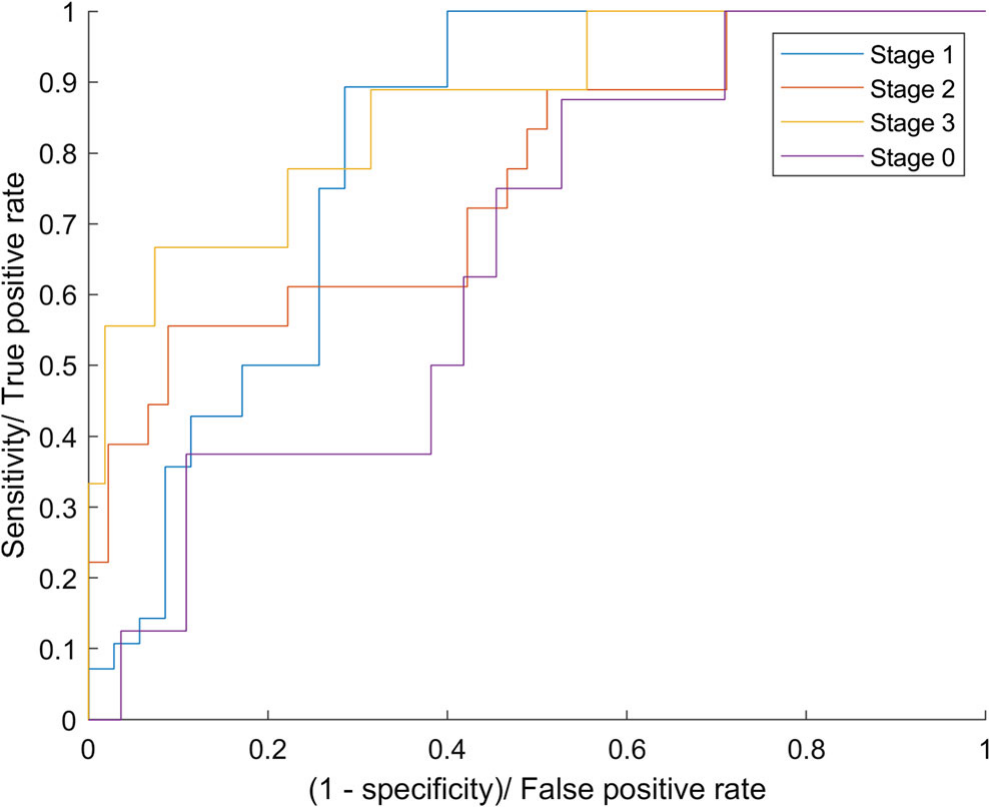
Receiver operating characteristics for the system combining ANN with HOG for all stages of cellulite severity. Blue line shows results for patients with mild cellulite (stage 1). Red line shows results for patients with moderate cellulite (stage 2). Yellow line shows results for patients with severe cellulite (stage 3). Purple line shows results for patients without cellulite (stage 0)

We found that ANN with combination of HOG performs best. Details of the performance of this combination are provided in Table 3, wherein miss rate (FNR) and fall out (FPR) parameters show the error information. Other values in Table 3 refer to the correctness of the classification model. We found best result for stage 1 with sensitivity 0.89 and specificity 0.65. For stage 2 and stage 3, the sensitivity was lower (0.55 and 0.33 respectively) although the specificity was high (0.80 and 0.98 respectively). The corresponding receiver operating characteristic for HOG and ANN is shown in Fig. 7.

**Table 3:** Summary of test data results for combination of ANN with HOG

Stage	Total test data	Sensitivity (TPR)	Miss rate (FNR)	Specificity (TNR)	Precision (PPV)	Fall out (FPR)	*F*-score	AUC	Class accuracy
Stage 0 (healthy)	8	0.125	0.875	0.963	0.333	0.036	0.181	0.656	0.857
Stage 1 (mild)	28	0.892	0.108	0.657	0.675	0.342	0.769	0.810	0.761
Stage 2 (moderate)	18	0.555	0.445	0.8	0.526	0.2	0.540	0.762	0.730
Stage 3 (severe)	9	0.333	0.667	0.981	0.75	0.018	0.461	0.866	0.888
Average	63	0.47669	0.5233	0.85057	0.57133	0.14943	0.48828	0.77406	0.80952

We can see that the average accuracy to classify all stages using our automatic diagnosis protocol is above 80%, which is satisfactory result when compared to cellulite stage classification at a clinical level.

## Discussion

### Related studies

The classification and detection of the stages of the cellulite using infrared thermographic image processing is gaining increased interest. For example, Bauer et al. [2] have demonstrated the efficacy of IR thermography for automatic diagnosis of the stages of cellulite. These authors used manual image preprocessing and feature extraction within a custom-designed classification framework. The quantitative methodology put forward in this work was able to classify cellulite based on thermographic images with high accuracy. Mazurkiewicz et al. [37] have extended this work with advanced machine learning algorithms. They used a growing bubble algorithm for extracting features of interest from the thermographic images. ANN algorithm was then applied for the classification of cellulite stages. In the classification scheme, a three-layer perceptron was used for the neural network. Also, normalized temperature ranges were used during the final classification of the cellulite severity stage.

Previously, Nkengne et al. [9] have provided a temperature-based cellulite severity measure from the infrared thermographic image of cellulite. They used the roughness parameter to evaluate the hot and cold points of the images. Their image was manually cropped and fully depended on the temperature-based analysis of the images. Wilczyński et al. [38] proposed a method for evaluating the severity of the cellulite based on mean gray-level co-occurrence matrix contrast of the infrared thermographic images of cellulite. They showed that traditionally used parameters of infrared analysis such as local maximum and minimum temperature or the median temperature are not useful in evaluation of cellulite severity. Table 4 presents a comparison of classification protocols based on morphological analysis reported in the present and previous studies, which has applied thermographic imaging in terms of resulting diagnostic accuracy for classification.

**Table 4:** The diagnostic accuracy of the cellulite severity stage classification based on morphological attributes of thermographic images reported in the literature

Author/year	Preprocessing/feature extraction	Classification algorithm	Total data in dataset (train/test)	Accuracy/result
Present study	Automatic/HOG	ANN	212 (149/63)	80.95%
Mazurkiewicz et al. 2018 [37]	Automatic/growing bubble algorithm	ANN	140 (126/14)	74%
Bauer et al. 2018 [2]	Manual/using ImageJ software	Manual classification	118 (59/59)	97.96%
Wilczyński et al. 2017 [38]	Manual/gray-level co-occurrence matrix	No classification of cellulite stages	40	Measure cellulite treatment effectiveness
Nkengneet et al. 2013 [9]	Manual/manually selected feature	No classification of cellulite stages	39	Influence of environmental and body-related factors measured

### Artificial intelligence application for automatic cellulite stage recognition

Most of currently used methods for the classification of cellulite are subjective and depend on the experts’ assessment. Infrared thermographic image processing can be used as an alternative way of evaluating and classifying the severity of cellulite; however, presently reported in the literature, methods are usually based on the analysis of temperature range not on morphological analysis of the thermographic images of cellulite. Our approach is based entirely on the morphological analysis of the infrared thermographic images of cellulite. This approach can be used for building commercial systems that could be cost-effective and used for personal diagnostics of cellulite without the help of clinicians or cosmetologists.

In addition, unlike the approaches previously reported in the literature, we propose a fully automated, computer-based diagnosis protocol. This fully automatic image processing and decision-making protocol allows much faster repeat testing and analysis, thus increasing the objectivity, reliability, and reproducibility of diagnosis. We employ this automatic, computer-based image processing protocol on clinical IR thermographic images obtained from 212 female volunteers of age ranging between 19 and 22 years and possessing cellulite of different stages which has been diagnosed a priori with use of the Nürnberger-Müller scale [2, 10, 15]. We then compare the accuracy of the automatic diagnosis with respect to the accuracy of the diagnosis as per current clinical protocol.

### Analysis of the achieved accuracy results

Within this experiment, we got very good diagnostic accuracy for stage 1 which may be used in early detection of cellulite and thus serve in the future as a preventive tool. This will minimize the cost of overall treatment, as well as may lead to better therapeutic results due to timely prophylactic intervention. Also the classification accuracy for all the other stages of cellulite is satisfactory (especially for stage 3 and stage 0); however, the sensitivity of the proposed method initially was unsatisfactory. To improve it, we carried out another experiment using a better balanced database which contained the same number of images of different stages of the cellulite. In the initial image database, there were 27 datasets for stage 0 and 31 datasets for stage 3 whereas stage 1 has maximum of 93 data (43% of total data) sets. This large portion of data could make the result biased. To check the influence of the data discrepancy, we reduced our data from stage 1 and stage 2 to the minimum data of 27 to achieve a balanced dataset. Then the system was trained and tested again with equal number of test data of each class. We found that the results for stage 0 and stage 3 provided better sensitivity, as both of these stages show the most discriminative features. These results are shown in supplementary materials (see Appendix 5: Table S9 and Fig. S1).

### Limitations of the study, potential for improvements, and recommendations with respect to PPPM

There is currently no in silico model for fully automatic recognition of cellulite severity which could be used as preventive tool and provide fully objective early diagnosis. We address this gap in our manuscript by developing and validating a computer-aided recognition system that combines custom-developed image preprocessing algorithm to automatically select cellulite regions with fully objective automated cellulite stage identification providing high accuracy. The proposed system allows women an early and reliable early diagnosis. It may also be used for monitoring of cellulite eventual progress in time. Potential treatment interventions and their outcomes can correspondingly be monitored.

For the purpose of this experiment, we used FLIR T335 camera with thermal sensitivity 50 mK and image resolution 320 × 240 pixels. Currently available on the market, FLIR cameras have comparable or superior thermal resolutions (usually < 30 mK at 30 °C) and either bigger IR detectors arrays (typically 640 × 480 and 1024 × 768) or UltraMax™ function built in that let to combine the information from multiple recorded original images into one image with approximately 50% less noise and superresolution (usually 4 times more pixels in output image in comparison to the size of focal plane array). Thermographic images provided by these cameras are larger and contain more details, as the single measurement spot size is smaller. This means that the overall accuracy of the potential recognition systems based on databases recorded by newest versions of FLIR cameras should be also higher, as they provide higher measurement accuracy to particularly small details and thus methods used for feature extraction will be able to possess more specific features for different stages of cellulite.

It should however be mentioned that working with cameras equipped with UltraMax™ system has also some limitations. For example, even in very stable mechanical conditions, e.g., that achieved by mounting the thermal imager on tripod, a good resolution picture may not result as the camera cannot capture pictures caused by small movements. Also, when there is too much movement, UltraMax™ function does not work well and output images may become blurred. In general, the UltraMax™ requires holding the camera with two hands during recording of thermographic images. Objects must be in focus. Also the contrast of the image must be high enough to let set of a thermogram to be aligned into one superresolution image.

On the contrary, the usage of small portable IR cameras, like those designed to be attached to mobile phones, may significantly decrease the accuracy of proposed recognition system. These IR cameras usually have small focal plane arrays (e.g., 80 × 60) and thermal sensitivity (e.g., < 100 mK). It is highly probable that the use of thermographic images with such a low resolution will not only cause a significant decrease in the accuracy of recognition system, but may also require the usage of a different combination of feature extraction methods and classifiers to obtain optimal results. That will require recording of new database and repeating both the training of classification algorithms with new learning databases and validation of classifiers with new test databases to find optimal combination.

Although the methodology proposed by us does not provide the 100% accuracy as a fully automatic protocol, the ability to recognize different cellulite stages is very promising for PPPM, especially for the purpose of predictive diagnosis. For PPPM, the method for detection and interpretation of skin patterns will have to be validated with respect to associated or similar conditions of cellulite. It is reasonable to anticipate that predictability performance of the methodology developed here will improve with the increase in the number of thermographic images for each stage and maintaining a good balance between the number of images used for training and testing for each degree. This will facilitate developments of both personalized intervention and potential prevention measures using specific data obtained from predictive diagnosis. Both prediction and personalized treatments of cellulite would have to be supported by trained professionals such as dermatologist or a cosmetologist. We are of the view that future further patients’ stratification and phenotyping will be possible, as well as providing an in-depth recognition of different types of cellulite like hard, soft, edematous, or mixed one or to stratify the different phenotypes. However, achieving that will require further extension and differentiation of database by the simultaneous application of various diagnostic tools (e.g., IR imaging combined with static and dynamic elastography, magnetic resonance imaging, computed tomography, videocapillaroscopy, high-frequency or Doppler ultrasonography). In addition, it will be necessary to link image data with detailed information about patients’ lifestyle, sports activity, ethnicity, eating habits, health state, e.g., overweight, underweight, metabolic disorders, undergoing rehabilitation, medications taken, etc. The implementation of AI and IR thermography in these areas would also require the continuation of the experiments as a function of time, because several studies on cellulites suggest that different types of cellulite are time related and dominate in women of different ages.

## Conclusions

Despite the widespread information about cellulite and its pathogenesis, there is still a shortage of fast and widely available tools for an objective analysis and early diagnosis of cellulite. This article shows that the implementation of the artificial intelligence for computer-aided, automatic recognition of cellulite stage based on non-contact IR imaging is feasible for the objective and reliable early diagnosis. This article thus contributes to the development of PPPM by providing a methodology for objective assessment of the cellulite stage, automation, and speed of the process which can provide cellulite assessment to virtually anyone who has access to internet and a suitable thermal camera. The combination of AI with thermographic diagnosis and decision-making can detect both initial and advanced cellulite stages and may be used by women for prescreening and early diagnosis without the help of clinicians or cosmetologists, for example, using IR camera attached to smart phones and dedicated “app.”

The combination approach of artificial intelligence with IR thermography can be adapted to many other relevant medical domains and facilitate implementation of PPPM. Our work creates the basis for more complex, larger studies towards more adequate patients’ stratification and phenotyping, as well as better understanding of skin pathophysiology through the use of thermography in predictive assessment of health status. Finding a correlation between IR imaging and pathological processes, while important, is currently difficult. Similarly challenging is the determination of the impact of personal and environmental factors (e.g., ethnicity, lifestyle, physical activity, eating habits, health state) on the thermographic imaging. The current study can be taken as a starting point for further elaboration and long-term investigations that would have to use a number of different diagnostic tools including, but not limited to, IR imaging for cross verification. These investigations must be carried out simultaneously on each volunteer and combined with detailed questionnaire to find out the impact of external and internal factors on physiological processes as to be detected in imaging. Furthermore, the experiment must consider the time domain as the types of cellulite (i.e., hard, soft, edematous, and mixed) are time related and dominate in women of different ages. In summary, while our work shows that IR imaging can detect cellulite, a correlation between imaging and cellulite pathogenesis requires far more complex, time consuming, cross-sectional, and cross-modality investigation in future.

This correlation with pathogenesis should constitute the primary, long-term vision of further development of PPPM for cellulites using IR thermography. Self-monitoring of cellulite progress and personalization of cellulite therapy by fast and effective evaluation of the treatment outcomes and therapeutic endpoint are two other directions that further development can adopt in the short and medium term for the applicability of IR thermography for the patients/individuals, as well as for the healthcare system in the global view. IR thermography coupled to AI in this way can develop as a robust tool for complex assessment of health status in the field of medical imaging and PPPM. The practical application of the method presented in this paper may speed up further development of PPPM and stimulate other experts in the field of thermography, artificial intelligence, and beyond to work on other target groups of patient as well as healthy individuals.

## Electronic supplementary material

ESM 1 (DOCX 125 kb).

## Abbreviations

ANN: Artificial neural network
AUC: Area under the curve
BRISK: Binary robust invariant scalable keypoints
Cont: Contour-based feature
DT: Decision tree
EucDist: Euclidean distance
FNR: False negative rate (miss rate)
FPR: False positive rate (fall out rate)
HOG: Histogram of oriented gradient
HOG_BRSIK: Combination of BRISK with HOG
ICT: Information and communication technologies
KNN: *K* nearest neighbor
LBP: Linear binary patterns
LDA: Linear discriminant analysis
LogReg: Logistic regression
MinDist: Minimum distance
NB: Naive Bayes
PCA: Principal component analysis
PPPM: Predictive, preventive, and personalized medicine
PPV: Positive predictive value (precision)
PR: Pattern recognition
RF: Random forest
ROC: Receiver operating characteristics
ROI: Region of interest
Stat: Statistical features of intensity value of the image
SURF: Speeded up robust feature
SVM: Support vector machine
TPR: True positive rate (sensitivity)
TNR: True negative rate (specificity)
