# Supplementary material for: Implementation of artificial intelligence and non-contact infrared thermography for prediction and personalized automatic identification of different stages of cellulite
Source: EPMA J. 2020 Feb 7;11(1):17–29. doi: 10.1007/s13167-020-00199-x (PMC7028894; doi:10.1007/s13167-020-00199-x)
Supplement: Supplementary file 1 — (DOCX 125 kb). [file 13167_2020_199_MOESM1_ESM.docx]

**SUPPLEMENTAL MATERIALS**

**APPENDIX 1:**

**Image Preprocessing Algorithm**

Below we present the pseudo code of the applied image preprocessing algorithm

**Preprocessing function (img):**

1. Adjust histogram of the image
2. Convert RGB image to YCbCr
3. Remove noise by dividing image into 10x10 grid and summation of color values
4. Identify edge using Sobel edge detection method
5. Dilate the edge and fill the area
6. Find label connected components from the image
7. Find the Region of the thigh as bounding box
8. Crop the selected bounding box region
9. Reshape the image to a constant size

**APPENDIX 2:**

**Feature Extraction Methods used**

1. **Euclidean distance (EucDist)**

Euclidean distance is widely used in image classification. In image processing Euclidean distance is the straight line distance between two pixels and this is evaluated using the following formula. If *P*(*x*_1_, *y*_1_) and *Q(x_2_, y_2_)* are the two pixels in an image then the Euclidean distance is

$$\boldsymbol{d}\left( \boldsymbol{p}, \boldsymbol{q} \right)= \sqrt{\left( X_{2}-X_{1} \right)^{2}+\left( Y_{2}-Y_{1} \right)^{2}}$$

We find the distance of two images using the summation of all distance using the following formula

$$\boldsymbol{OverallDistance}= \sum_{\mathbf{i=1}}^{\boldsymbol{n}} \left( p_{i}-q_{i} \right)^{2}$$

Where P_i_ is the itch pixel of one image and Q_i_ is the itch pixel of another image.
We find the Euclidean distance for each test image from all the training images. We build a matrix of Euclidean distance where in each row there are test images and each column indicates the train image. Then we find minimum distance from each test image to all train images. The test image tends to be more likely to the minimum distanced train images class.

1. **Statistical feature (Stat):**

As statistical features we considered i.e. mean, variance, skewness, kurtosis, entropy and energy of the intensity value of the image.

- **Mean**

For a random variable vector A made up of N scalar observations [1]. The mean is defined as

*μ* (Mean) = $\frac{Summation of intesity Value}{Total number of intensity}$ = $\frac{1}{N}\sum_{k=1}^{N} A_{k}$

- **Variance**

For a random variable vector A made up of N scalar observations, the variance is defined as

σ (Variance) = $\frac{1}{N-1}\sum_{k=1}^{N} {(A_{k}- \mu)}^{2}$

Where μ is the mean of A,

- **Skewness**:

Skewness is a measure of the asymmetry of the data around the sample mean [2]. If skewness is negative, the data are spread out more to the left of the mean than to the right. If skewness is positive, the data are spread out more to the right. The skewness of the normal distribution (or any perfectly symmetric distribution) is zero.

The skewness of a distribution is defined as

s (Skewness) = $\frac{E{(x- \mu)}^{3}}{\sigma^{3}}$

Where µ is the mean of x, σ is the standard deviation of x, and E(x) represents the expected value of the variable x.

- **Kurtosis**:

Kurtosis is a measure of how outlier-prone a distribution is [3]. The kurtosis of the normal distribution is 3. Distributions that are more outlier-prone than the normal distribution have kurtosis greater than 3; distributions that are less outlier-prone have kurtosis less than 3. The kurtosis of a distribution is defined as

*K (Kurtosis)* = $\frac{E{(x- \mu)}^{4}}{\sigma^{4}}$

- **Entropy**

Entropy is a statistical measure of randomness that can be used to characterize the texture of the input image [4]. Entropy is defined as

*Entropy = − sum(p×log_2_(p))*, where p contains the normalized histogram counts returned from histogram value of the image.

- **Energy**

We calculate energy using the following formula

E=$\frac{1}{(M*N)}\sum_{k=1}^{MN} {(I_{k})}^{2}$

Where I is an image and M is the number of row of the image and N is the number of column of the image and $I_{k}$represents the intensity value of each element of image.

1. **Contour based feature (Cont)**

Contour is a curve along which the function has a constant value so that the curve joins points of equal value [5]. There are two kinds of contour: open contour and closed contour. In case of cellulite the contours of different shapes are created. With the changes of cellulite Stage the contour property changed. We consider the following properties of contours of cellulite image:

1. No of contour in a cellulite image
2. No of closed contour in the image
3. Sum of the area of all contour line
4. Sum of the length of all contour line in the image
5. Sum of levels of the contour in the image
6. **Principal Component Analysis (PCA)**

The principal component analysis is a technique for feature extraction which combines input variables in a specific way that let to drop the “least important” variables while still retaining the most valuable parts of all of the variables [6]. PCA finds lower dimensional surface so that the sum of square projection error is minimized. The property of the maximum variation of the projected points defines the first principal axis, it is the line or direction with the maximum variation of the projected values of the original data points. The projection values corresponding to this direction of maximum variation are the principal component score. The first principal component also minimizes the total distance between the data and their projection onto the principal component axes, and moreover maximize the variance of projected points. The rest of the principal components are also selected similarly but to make sure that these are uncorrelated (orthogonal) with all previous principal components [7].

1. **Histogram of Oriented Gradient (HOG)**

Histogram of oriented gradient (HOG) is a feature descriptor which is extensively used in computer vision and image processing for the purpose of object detection and recognition. In HOG, the distribution (histograms) of directions of gradients (oriented gradients) are used as the feature. Gradients are *x* and *y* derivatives of an image. Gradient is useful in image processing because the magnitude of gradients is large around edges and corners. HOG used filtering (Sobel or others) for finding gradients magnitude and direction in horizontal & vertical directions. Finally, HOG feature detector encodes local shape information from regions within an image. MATLAB has *extractHOGFeatures(img, 'CellSize'* method for finding HOG features where *img* refers to the image.

1. **Linear Binary Patterns (LBP)**

LBP features encode local texture information, which can be used for tasks such as classification, detection, and recognition. Ojala et al first published a paper on LBP [8]. LBP are fundamental properties of local image texture and their occurrence histogram proved very powerful texture feature. It is invariant to gray-scale and rotation. It is mainly used for texture-based image classification. LBP simply divide the image into cells. Then based on neighbor pixel we compute the histogram over the cell, normalize the histogram and finally concatenate the histograms of all cells that gives the feature vector. MATLAB has *extractLBPFeatures*(*img,'CellSize',* method for finding LBP features where *img* refers to the image.

1. **Speeded Up Robust Feature (SURF)**

SURF is a feature detection algorithm which is mainly used in image classification, image registration, and image matching. SURF is an updated version of SIFT (Scale-Invariant Feature Transform) algorithm [9]. SURF mainly work with image blob (is a region of an image in which some properties are constant or approximately constant). For orientation assignment, SURF uses wavelet responses in horizontal and vertical direction for a neighborhood of size 6s. SURF works well even with image blurring and rotation. But it cannot handle viewpoint change and illumination change. MATLAB has detectSURFFeatures(I) and extractFeatures(I, points) method for finding SURF features where I refers to the Image and points returned by the *detectSURFFeatures (I)* methods.

1. **Binary Robust Invariant Scalable Keypoints (BRISK)**

Binary Robust Invariant Scalable Keypoints (BRISK) is a feature descriptor algorithm for keypoint detection, description and matching. It is based on scale-space FAST based detector. BRISK is faster and it incurs low computational cost [10]. BRISK detects multiscale corner features which is used in image classification, image matching and image registration. MATLAB has detectBRISKFeatures(I) and extractFeatures(I, points) method for finding BRISK features where I refers to the Image and points returned by the detectBRISKFeatures(I) methods.

1. **Combination of BRISK and HOG (BRISK_HOG)**

Finally we first used BRISK to detect the points and then apply HOG on detected point to find the feature of cellulite image. After implementing detectBRISKFeatures(I) methods we have implemented extractHOGFeatures(I, points, 'CellSize', methods where points are returned by the detectBRISKFeatures(I) methods.

**APPENDIX 3:**

**Performance of different Feature Extraction methods combinations with Artificial Neural Network (ANN)**

Table S1: Accuracy of different combinations of Feature Extraction Methods with ANN

| Feature Extraction Method | Accuracy Stage 1 | Accuracy Stage 2 | Accuracy Stage 3 | Accuracy Stage 0 | Average Accuracy |
| --- | --- | --- | --- | --- | --- |
| PCA | 0.63492 | 0.65079 | 0.85714 | 0.87302 | 0.75397 |
| EucDist | 0.50794 | 0.61905 | 0.79365 | 0.87302 | 0.69841 |
| Stat | 0.39683 | 0.65079 | 0.85714 | 0.85714 | 0.69048 |
| Cont | 0.69841 | 0.57143 | 0.79365 | 0.85714 | 0.73016 |
| HOG | 0.76190 | 0.73016 | 0.88889 | 0.85714 | 0.80952 |
| LBP | 0.66667 | 0.63492 | 0.90476 | 0.84127 | 0.76190 |
| SURF | 0.52381 | 0.60317 | 0.84127 | 0.88889 | 0.71429 |
| BRISK | 0.60317 | 0.5873 | 0.80952 | 0.7619 | 0.69048 |
| HOG_BRISK | 0.5873 | 0.65079 | 0.84127 | 0.80952 | 0.72222 |

Legend: PCA - Principal Components Analysis, EucDist - Euclidean Distance, Stat - Statistical features of intensity value of the image (mean, variance, skewness etc.), Cont - Contour based feature (length of contour, area of contour etc.), HOG - Histogram of Oriented Gradient, LBP -Linear Binary Patterns, SURF - Speeded Up Robust Feature, BRISK - Binary Robust Invariant Scalable Keypoints, HOG_BRSIK - combination of BRISK with HOG, ANN - Artificial Neural Network

Table S2: Average areas under curve (AUC) of different combinations of Feature Extraction Methods with Artificial Neural Network ANN

| Feature Extraction Method | AUC Stage 1 | AUC Stage 2 | AUC Stage 3 | AUC Stage 0 | Average AUC |
| --- | --- | --- | --- | --- | --- |
| PCA | 0.64694 | 0.61728 | 0.47942 | 0.63182 | 0.59387 |
| EucDist | 0.50714 | 0.58333 | 0.55556 | 0.50000 | 0.53651 |
| Stat | 0.50408 | 0.50247 | 0.52263 | 0.70455 | 0.55843 |
| Cont | 0.74796 | 0.55679 | 0.63992 | 0.79318 | 0.68446 |
| HOG | 0.81020 | 0.76296 | 0.86626 | 0.65682 | 0.77406 |
| LBP | 0.69082 | 0.67778 | 0.7716 | 0.67045 | 0.70266 |
| SURF | 0.54592 | 0.51235 | 0.77984 | 0.87045 | 0.67714 |
| BRISK | 0.57245 | 0.59630 | 0.6070 | 0.51136 | 0.57178 |
| HOG_BRISK | 0.65510 | 0.52222 | 0.61523 | 0.66591 | 0.61461 |

Legend: PCA - Principal Components Analysis, EucDist - Euclidean Distance, Stat - Statistical features of intensity value of the image (mean, variance, skewness etc.), Cont - Contour based feature (length of contour, area of contour etc.), HOG - Histogram of Oriented Gradient, LBP -Linear Binary Patterns, SURF - Speeded Up Robust Feature, BRISK - Binary Robust Invariant Scalable Keypoints, HOG_BRSIK - combination of BRISK with HOG, ANN - Artificial Neural Network

Table S3: Specificity of different combinations of Feature Extraction Methods with ANN

| Feature Extraction  Method | Specificity Stage 1 | Specificity Stage 2 | Specificity Stage 3 | Specificity Stage 0 | Average Specificity |
| --- | --- | --- | --- | --- | --- |
| PCA | 0.42857 | 0.75556 | 1,00000 | 1,00000 | 0.79603 |
| EucDist | 0.51429 | 0.66667 | 0.88889 | 1,00000 | 0.76746 |
| Stat | 0.08571 | 0.80889 | 1,00000 | 0.96364 | 0.73456 |
| Cont | 0.71429 | 0.62222 | 0.90741 | 0.96364 | 0.80189 |
| HOG | 0.65714 | 0.80000 | 0.98148 | 0.96364 | 0.85057 |
| LBP | 0.65714 | 0.66667 | 0.98148 | 0.96364 | 0.81723 |
| SURF | 0.31429 | 0.75556 | 0.98148 | 1,00000 | 0.76283 |
| BRISK | 0.74286 | 0.62222 | 0.90741 | 0.85455 | 0.78176 |
| HOG_BRISK | 0.57143 | 0.75556 | 0.98148 | 1,00000 | 0.82712 |

Legend: PCA - Principal Components Analysis, EucDist - Euclidean Distance, Stat - Statistical features of intensity value of the image (mean, variance, skewness etc.), Cont - Contour based feature (length of contour, area of contour etc.), HOG - Histogram of Oriented Gradient, LBP -Linear Binary Patterns, SURF - Speeded Up Robust Feature, BRISK - Binary Robust Invariant Scalable Keypoints, HOG_BRSIK - combination of BRISK with HOG, ANN - Artificial Neural Network

Table S4: Sensitivity of different combinations of Feature Extraction Methods with ANN

| Feature Extraction  Method | Sensitivity Stage 1 | Sensitivity Stage 2 | Sensitivity Stage 3 | Sensitivity Stage 0 | Average Sensitivity |
| --- | --- | --- | --- | --- | --- |
| PCA | 0.89286 | 0.38889 | 0,00000 | 0,00000 | 0.32044 |
| EucDist | 0.50000 | 0.50000 | 0.22222 | 0,00000 | 0.30556 |
| Stat | 0.78571 | 0.05555 | 0,00000 | 0.12500 | 0.24157 |
| Cont | 0.67857 | 0.44444 | 0.11111 | 0.12500 | 0.33978 |
| HOG | 0.89286 | 0.55556 | 0.33333 | 0.12500 | 0.47669 |
| LBP | 0.67857 | 0.55556 | 0.44444 | 0,00000 | 0.41964 |
| SURF | 0.78571 | 0.22222 | 0,00000 | 0.12500 | 0.28323 |
| BRISK | 0.42857 | 0.50000 | 0.22222 | 0.12500 | 0.31895 |
| HOG_BRISK | 0.78571 | 0.22222 | 0.11111 | 0.12500 | 0.31101 |

Legend: PCA - Principal Components Analysis, EucDist - Euclidean Distance, Stat - Statistical features of intensity value of the image (mean, variance, skewness etc.), Cont - Contour based feature (length of contour, area of contour etc.), HOG - Histogram of Oriented Gradient, LBP -Linear Binary Patterns, SURF - Speeded Up Robust Feature, BRISK - Binary Robust Invariant Scalable Keypoints, HOG_BRSIK - combination of BRISK with HOG, ANN - Artificial Neural Network

**APPENDIX 4:**

**Performance of HOG combination with different Classification Algorithms**

Table S5: Accuracy of different combinations of Classification Algorithms with HOG

| Classification Algorithm | Accuracy Stage 1 | Accuracy Stage 2 | Accuracy Stage 3 | Accuracy Stage 0 | Average Accuracy |
| --- | --- | --- | --- | --- | --- |
| MinDist | 0.50794 | 0.61905 | 0.79365 | 0.87302 | 0.69841 |
| KNN | 0.69841 | 0.63492 | 0.87302 | 0.80952 | 0.75397 |
| NB | 0.65079 | 0.69841 | 0.85714 | 0.80952 | 0.75397 |
| LogReg | 0.71429 | 0.66667 | 0.88889 | 0.84127 | 0.77778 |
| SVM | 0.71429 | 0.66667 | 0.88889 | 0.84127 | 0.77778 |
| LDA | 0.66667 | 0.65079 | 0.88889 | 0.80952 | 0.75397 |
| DT | 0.58730 | 0.68254 | 0.88889 | 0.76190 | 0.73016 |
| RF | 0.63492 | 0.71429 | 0.88889 | 0.90476 | 0.78571 |
| ANN | 0.76190 | 0.73016 | 0.88889 | 0.85714 | 0.80952 |

Legend: Minimum Distance (MinDist), K Nearest Neighbor (KNN), Naive Bayes (NB), Logistic Regression (LogReg), Support Vector Machine (SVM), Linear Discriminant Analysis (LDA), Decision Tree (DT), Random Forest (RF) and Artificial Neural Network (ANN), HOG - Histogram of Oriented Gradient

Table S6: Average areas under curve (AUC) of different combinations of Classification Algorithms with HOG

| Classification Algorithm | AUC Stage 1 | AUC Stage 2 | AUC Stage 3 | AUC Stage 0 | Average AUC |
| --- | --- | --- | --- | --- | --- |
| MinDist | 0.50714 | 0.58333 | 0.55556 | 0.50000 | 0.53651 |
| KNN | 0.69643 | 0.61111 | 0.64815 | 0.46364 | 0.60483 |
| NB | 0.65357 | 0.57222 | 0.73148 | 0.62386 | 0.64528 |
| LogReg | 0.80000 | 0.63333 | 0.85802 | 0.77500 | 0.76659 |
| SVM | 0.80000 | 0.63333 | 0.85802 | 0.77500 | 0.76659 |
| LDA | 0.66429 | 0.60556 | 0.7037 | 0.51705 | 0.62265 |
| DT | 0.58571 | 0.62778 | 0.61111 | 0.54318 | 0.59195 |
| RF | 0.66786 | 0.56667 | 0.65741 | 0.62500 | 0.62923 |
| ANN | 0.81020 | 0.76296 | 0.86626 | 0.65682 | 0.77406 |

Legend: Minimum Distance (MinDist), K Nearest Neighbor (KNN), Naive Bayes (NB), Logistic Regression (LogReg), Support Vector Machine (SVM), Linear Discriminant Analysis (LDA), Decision Tree (DT), Random Forest (RF) and Artificial Neural Network (ANN), HOG - Histogram of Oriented Gradient

Table S7: Specificity of different combinations of Classification Algorithms with HOG

| Classification Algorithm | Specificity Stage 1 | Specificity Stage 2 | Specificity Stage 3 | Specificity Stage 0 | Average Specificity |
| --- | --- | --- | --- | --- | --- |
| MinDist | 0.51429 | 0.66667 | 0.88889 | 1.00000 | 0.76746 |
| KNN | 0.71429 | 0.66667 | 0.96296 | 0.92727 | 0.8178 |
| NB | 0.62857 | 0.86667 | 0.90741 | 0.87273 | 0.81884 |
| LogReg | 0.65714 | 0.75556 | 0.96296 | 0.94545 | 0.83028 |
| SVM | 0.65714 | 0.75556 | 0.96296 | 0.94545 | 0.83028 |
| LDA | 0.68571 | 0.71111 | 0.96296 | 0.90909 | 0.81722 |
| DT | 0.60000 | 0.75556 | 1.00000 | 0.83636 | 0.79798 |
| RF | 0.37143 | 0.91111 | 0.98148 | 1.00000 | 0.81601 |
| ANN | 0.65714 | 0.80000 | 0.98148 | 0.96364 | 0.85057 |

Legend: Minimum Distance (MinDist), K Nearest Neighbor (KNN), Naive Bayes (NB), Logistic Regression (LogReg), Support Vector Machine (SVM), Linear Discriminant Analysis (LDA), Decision Tree (DT), Random Forest (RF) and Artificial Neural Network (ANN), HOG - Histogram of Oriented Gradient

Table S8: Sensitivity of different combinations of Classification Algorithms with HOG

| Classification Algorithm | Sensitivity Stage 1 | Sensitivity Stage 2 | Sensitivity Stage 3 | Sensitivity Stage 0 | Average Sensitivity |
| --- | --- | --- | --- | --- | --- |
| MinDist | 0.50000 | 0.50000 | 0.22222 | 0,00000 | 0.30556 |
| KNN | 0.67857 | 0.55556 | 0.33333 | 0,00000 | 0.39187 |
| NB | 0.67857 | 0.27778 | 0.55556 | 0.37500 | 0.47173 |
| LogReg | 0.78571 | 0.44444 | 0.44444 | 0.12500 | 0.44990 |
| SVM | 0.78571 | 0.44444 | 0.44444 | 0.12500 | 0.44990 |
| LDA | 0.64286 | 0.50000 | 0.44444 | 0.125000 | 0.42808 |
| DT | 0.57143 | 0.50000 | 0.22222 | 0.25000 | 0.38591 |
| RF | 0.86429 | 0.22222 | 0.33333 | 0.25 | 0.44246 |
| ANN | 0.89286 | 0.55556 | 0.33333 | 0.125 | 0.47669 |

Legend: Minimum Distance (MinDist), K Nearest Neighbor (KNN), Naive Bayes (NB), Logistic Regression (LogReg), Support Vector Machine (SVM), Linear Discriminant Analysis (LDA), Decision Tree (DT), Random Forest (RF) and Artificial Neural Network (ANN), HOG - Histogram of Oriented Gradient

**APPENDIX 5:**

**Performance results of combination of HOG and ANN for balanced Dataset with equal number of images for different stages of the cellulite**

Table S9: Results for balanced dataset for combination of HOG with ANN

| Stage | Total Test data | Sensitivity (TPR) | Miss Rate (FNR) | Specificity (TNR) | Precision (PPV) | Fall out (FPR) | F-score | AUC | Class Accuracy |
| --- | --- | --- | --- | --- | --- | --- | --- | --- | --- |
| Stage 0 (Healthy) | 8 | 0.75000 | 0.2500 | 0.75000 | 0.46131 | 0.250000 | 0.600000 | 0.864583 | 0.7500 |
| Stage 1 (mild) | 8 | 0.37500 | 0.6250 | 0.83333 | 0.42857 | 0.166667 | 0.400000 | 0.723958 | 0.71875 |
| Stage 2 (moderate) | 8 | 0.12500 | 0.8750 | 0.87500 | 0.25000 | 125000 | 0.400000 | 0.640625 | 0.6875 |
| Stage 3 (severe) | 8 | 0.75000 | 0.2500 | 0.87500 | 0.66666 | 125000 | 0.705882 | 0.880208 | 0.84375 |
| Average of all Stages | 32 | 0.50000 | 0.500 | 0.83333 | 0.50000 | 0.461310 | 0.468137 | 0.777344 | 0.7500 |


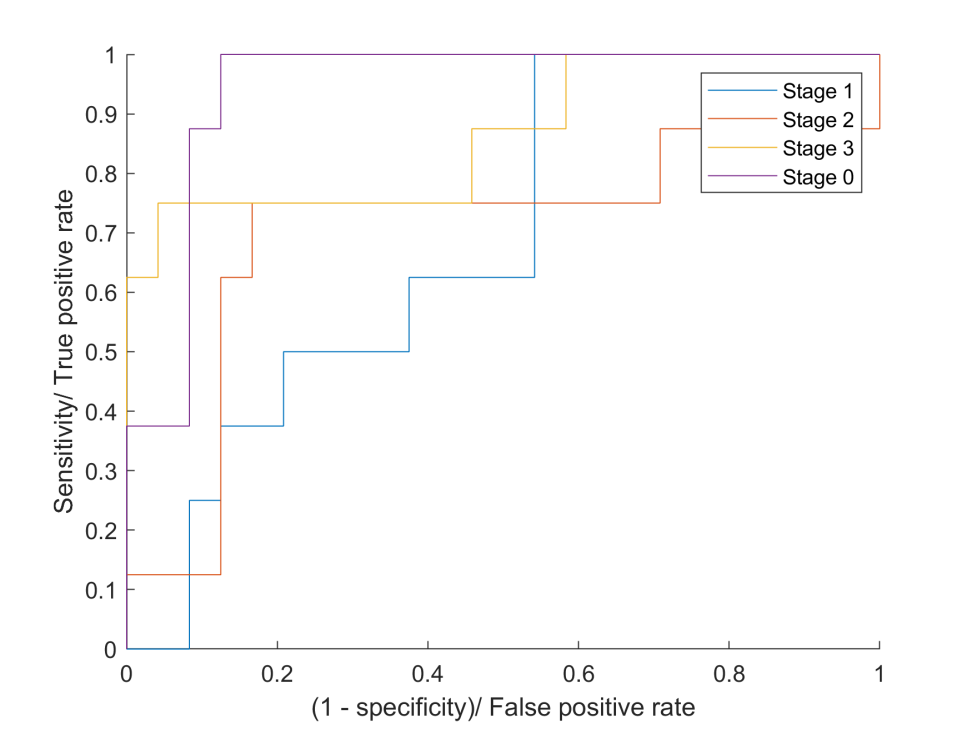


Figure S1: Performance of test data with balanced dataset

**References**

| [1] Average or mean. 2018. https://uk.mathworks.com/help/matlab/ref/mean.html. Accessed 09 Oct 2018. |
| --- |
| [2] Skewness. 2018. https://uk.mathworks.com/help/stats/skewness.html. Accessed 09 Oct 2018. |
| [3] Kurtosis. 2018. https://uk.mathworks.com/help/stats/kurtosis.html. Accessed 09 Oct 2018. |
| [4] Entropy. 2018. https://uk.mathworks.com/help/images/ref/entropy.html. Accessed 09 Oct 2018. |
| [5] Contour line. 2018. https://en.wikipedia.org/wiki/Contour_line. Accessed 09 Oct 2018. |
| [6] Principal Component Analysis. 2018. https://www.dezyre.com/data-science-in-python-tutorial/principal-component-analysis-tutorial. Accessed 09 Oct 2018. |
| [7] Lever J, Krzywinski M, Altman N. Points of significance: Principal component analysis. Nature Methods 2017;14(7):641−2. |
| [8] Ojala T, Pietikainen M, Maenpaa T. Multiresolution gray-scale and rotation invariant texture classification with local binary patterns. IEEE Trans Pattern Anal Mach Intell 2002; 24(7):971−87. |
| [9] Lowe DG. Distinctive image features from scale-invariant keypoints. Int J Computer Vis 2004;60(2):91−110. |
| [10] Leutenegger S, Chli M, Siegwart RY. BRISK: Binary robust invariant scalable keypoints. In: Proceedings of IEEE International Conference on Computer Vision. 2011. pp. 2548−55. |
